# Progressive right ventricular dysfunction and exercise impairment in patients with heart failure and diabetes mellitus: insights from the T.O.S.CA. Registry

**DOI:** 10.1186/s12933-022-01543-3

**Published:** 2022-06-16

**Authors:** Andrea Salzano, Roberta D’Assante, Massimo Iacoviello, Vincenzo Triggiani, Giuseppe Rengo, Francesco Cacciatore, Ciro Maiello, Giuseppe Limongelli, Daniele Masarone, Angela Sciacqua, Pasquale Perrone Filardi, Antonio Mancini, Maurizio Volterrani, Olga Vriz, Roberto Castello, Andrea Passantino, Michela Campo, Pietro A. Modesti, Alfredo De Giorgi, Michele Arcopinto, Paola Gargiulo, Maria Perticone, Annamaria Colao, Salvatore Milano, Agnese Garavaglia, Raffaele Napoli, Toru Suzuki, Eduardo Bossone, Alberto M. Marra, Antonio Cittadini, T.O.S.CA. Investigators, A. Cittadini, A. M. Marra, M. Arcopinto, R. D’Assante, L. Saccà, M. G. Monti, R. Napoli, M. Matarazzo, F. M. Stagnaro, L. Piccioli, A. Lombardi, V. Panicara, M. Flora, L. Golia, V. Faga, A. Ruocco, D. Della Polla, R. Franco, A. Schiavo, A. Gigante, E. Spina, M. Sicuranza, F. Monaco, M. Apicella, C. Miele, A. G. Campanino, L. Mazza, R. Abete, A. Farro, F. Luciano, R. Polizzi, G. Ferrillo, M. De Luca, G. Crisci, F. Giardino, M. Barbato, A. Salzano, B. Ranieri, E. Bossone, F. Ferrara, V. Russo, M. Malinconico, R. Citro, E. Guastalamacchia, M. Iacoviello, M. Leone, V. Triggiani, V. A. Giagulli, F. Cacciatore, C. Maiello, C. Amarelli, I. Mattucci, G. Limongelli, D. Masarone, P. Calabrò, R. Calabrò, A. D’Andrea, V. Maddaloni, G. Pacileo, R. Scarafile, F. Perticone, A. Belfiore, A. Sciacqua, A. Cimellaro, P. Perrone Filardi, L. Casaretti, S. Paolillo, P. Gargiulo, A. Mancini, A. M. R. Favuzzi, C. Di Segni, C. Bruno, E. Vergani, M. Volterrani, R. Massaro, O. Vriz, F. Grimaldi, R. Castello, A. Frigo, M. R. Campo, M. R. Sorrentino, P. A. Modesti, D. Malandrino, R. Manfredini, A. De Giorgi, F. Fabbian, A. Puzzo, L. Ragusa, L. Caliendo, L. Carbone, A. Frigiola, T. Generali, F. Giacomazzi, C. De Vincentiis, A. Ballotta, P. Garofalo, G. Malizia, S. Milano, G. Misiano, T. Suzuki, M. Z. Israr, D. Bernieh, S. Cassambai, Y. Yazaki, L. M. Heaney, K. A. Eagle, H. O. Ventura, A. Colao, D. Bruzzese

**Affiliations:** 1 IRCCS SYNLAB SDN, Diagnostic and Nuclear Research Institute, Naples, Italy; 2 Department of Translational Medical Sciences, Federico II University, Naples, Italy; 3 Italian Clinical Outcome Research and Reporting Program (I-CORRP), Naples, Italy; 4 Cardiology Unit, Department of Medical and Surgical Sciences, University of Foggia, 71122 Foggia, Italy; 5 Interdisciplinary Department of Medicine-Section of Internal Medicine, Geriatrics, Endocrinology and Rare Diseases, University of Bari ‘A Moro’, Bari, Italy; 6 Istituti Clinici Scientifici Maugeri SpA Società Benefit (ICS Maugeri SpA SB) - IRCCS - Scientific Institute of Telese Terme, Telese Terme, BN Italy; 7 Heart Transplantation Unit, Monaldi Hospital, Azienda Ospedaliera Dei Colli, Naples, Italy; 8 Division of Cardiology, Monaldi Hospital, Azienda Ospedaliera Dei Colli, University of Campania Luigi Vanvitelli, Caserta, Italy; 9 Department of Medical and Surgical Sciences, University Magna Græcia of Catanzaro, Catanzaro, Italy; 10 Department of Advanced Biomedical Sciences, Federico II University, Naples, Italy; 11 Mediterranea Cardiocentro, Naples, Italy; 12 Operative Unit of Endocrinology, Catholic University of the Sacred Heart, Rome, Italy; 13 Department of Medical Sciences, IRCCS San Raffaele Pisana, Rome, Italy; 14 Heart Center Department, King Faisal Hospital & Research Center, Riyadh, Kingdom of Saudi Arabia; 15 Division of General Medicine, Azienda Ospedaliera Universitaria Integrata, Verona, Italy; 16 Scientific Clinical Institutes Maugeri, IRCCS, Pavia, Italy; 17 Unit of Endocrinology and Metabolic Diseases, Department of Medical and Surgical Sciences, University of Foggia, Foggia, Italy; 18 Dipartimento Di Medicina Sperimentale E Clinica, Università Degli Studi Di Firenze, Florence, Italy; 19 Department of Medical Sciences, School of Medicine, Pharmacy and Prevention, University of Ferrara, Ferrara, Italy; 20 Clinical Medicine and Surgery Department, Federico II University, Naples, Italy; 21 Department of Laboratory Medicine, AOUP P. Giaccone, Palermo, Italy; 22 Medical Department, Novartis Farma S.P.A., Origgio, Italy; 23 Department of Cardiovascular Sciences and NIHR Leicester Biomedical Research Centre, Glenfield Hospital, University of Leicester, Leicester, UK; 24 Cardiology Division, A Cardarelli Hospital, Naples, Italy; 25 Center for Pulmonary Hypertension, Thoraxclinic at Heidelberg University Hospital, Heidelberg, Germany; 26 Department of Translational Medical Sciences, Division of Internal Medicine and Metabolism and Rehabilitation, Federico II University of Naples, Via S. Pansini 5, Bld.18, 1stfloor, 80131 Naples, Italy

**Keywords:** Chronic heart failure, TOSCA, Diabetes, Insulin resistance, Cardiopulmonary exercise test, Right ventricle

## Abstract

**Background:**

Findings from the T.O.S.CA. Registry recently reported that patients with concomitant chronic heart failure (CHF) and impairment of insulin axis (either insulin resistance—IR or diabetes mellitus—T2D) display increased morbidity and mortality. However, little information is available on the relative impact of IR and T2D on cardiac structure and function, cardiopulmonary performance, and their longitudinal changes in CHF.

**Methods:**

Patients enrolled in the T.O.S.CA. Registry performed echocardiography and cardiopulmonary exercise test at baseline and at a patient-average follow-up of 36 months. Patients were divided into three groups based on the degree of insulin impairment: euglycemic without IR (EU), euglycemic with IR (IR), and T2D.

**Results:**

Compared with EU and IR, T2D was associated with increased filling pressures (E/e′ratio: 15.9 ± 8.9, 12.0 ± 6.5, and 14.5 ± 8.1 respectively, p < 0.01) and worse right ventricular(RV)-arterial uncoupling (RVAUC) (TAPSE/PASP ratio 0.52 ± 0.2, 0.6 ± 0.3, and 0.6 ± 0.3 in T2D, EU and IR, respectively, p < 0.05). Likewise, impairment in peak oxygen consumption (peak VO_2_) in TD2 vs EU and IR patients was recorded (respectively, 15.8 ± 3.8 ml/Kg/min, 18.4 ± 4.3 ml/Kg/min and 16.5 ± 4.3 ml/Kg/min, p < 0.003). Longitudinal data demonstrated higher deterioration of RVAUC, RV dimension, and peak VO_2_ in the T2D group (+ 13% increase in RV dimension, − 21% decline in TAPSE/PAPS ratio and − 20% decrease in peak VO_2_).

**Conclusion:**

The higher risk of death and CV hospitalizations exhibited by HF-T2D patients in the T.O.S.CA. Registry is associated with progressive RV ventricular dysfunction and exercise impairment when compared to euglycemic CHF patients, supporting the pivotal importance of hyperglycaemia and right chambers in HF prognosis.

*Trial registration* ClinicalTrials.gov identifier: NCT023358017

## Background

There is an intimate link between heart failure (HF) and type 2 diabetes (T2D) [1–6]. Both diseases share common pathophysiological mechanisms including insulin-resistance (IR) and neuro-hormonal activation. They often overlap and each disease increases the risk for the other. Indeed, the prevalence of T2D in HF cohort ranges from 10 to 47%, higher in hospitalized patients, while the prevalence of HF in T2D is 4 times higher than the general population ranging from 9 to 22%. On the other hand, IR represents a complex pathological condition that shapes the natural history of diabetes, influence its prognosis, and is strongly associated with its future development, accounting for up to 60% of patients with HF [4]. Importantly, while the effects of IR on HF outcomes are unclear, several community-based and hospitalized cohorts consistently showed increased risk of death and hospitalization in HF patients with T2D vs. euglycemic (EU) patients [4]. Likewise, many multivariate risk models highlight T2D as an independent risk factor for death in HF [7, 8]. T2D and IR may influence HF progression through several putative mechanisms, including metabolic inflexibility, impaired calcium handling, mitochondrial dysfunction, oxidative stress, and dysregulated myocardial-endothelial interactions. IR and hyperinsulinemia are thought to induce the so-called diabetic cardiomyopathy characterized, among others, by left ventricle (LV) hypertrophy and diastolic dysfunction predicts worsening LV function and remodeling [9]. On the other hand, T2D is characterized by hyperglycemia associated with IR. However, it is unclear whether the higher mortality observed in patients with diabetes and HF is due to hyperglycaemia per se or whether the presence of IR is already capable of affecting the HF progression. In this regard, data from the TOSCA Registry [10, 11] recently confirmed that the presence of insulin impairment (IR defined by HOMA index > 2.5 or T2D) was significantly associated with CV hospitalization and all-cause mortality. Of note, when adjusted for confounders, T2D alone and not HOMA-IR predicted outcome whereas HOMA-IR alone.

However, little information is available on the relative impact of IR and T2D on LV architecture and function as well as on cardiopulmonary performance, in HF cohorts. More importantly, no study dwelled upon the independent effect of IR or T2D on longitudinal changes of left and right chamber architecture and function, their coupling with the pulmonary circulation as well as cardiopulmonary. Therefore, the aims of the present investigation were to explore the separate impact of T2D and IR on left and right chambers morphology and function and cardiopulmonary performance and their longitudinal changes in relation to the cardiovascular outcomes.

## Methods

### Study population

The study design has been previously described [10–12]. In brief, the T.O.S.CA. Registry represents a prospective multicentre observational study, enrolling consecutive patients with stable CHF and left ventricular ejection fraction (LVEF) < 45%; inclusion criteria are as follows: no history of recent acute decompensation, acute coronary syndrome (< 6 months), severe liver (Cirrhosis Child-Turcotte-Pugh B-C), and/or kidney disease (creatinine level > 2.5 mg/dl) or active malignancy; further, patients need to be on stable medications for at least 3 months, including any beta-blocker (started at least 6 months before entering the study). As exclusion criteria, patients with history of current hormonal treatment or overt endocrine diseases were excluded.

### Study outcomes

For cross-sectional data, we considered as primary endpoints differences in echocardiographic and cardiopulmonary exercise test parameters comparing 3 groups: patients without IR impairment (EU), IR patients, and T2D patients.

With regard to longitudinal data, we compared the delta change (expressed as absolute values or percentages, as more suitable) in left ventricular dimension and function (i.e., ejection fraction and left ventricle end-diastolic volume), RV dimension, function, and RV to pulmonary arterial uncoupling (RVPUC) (i.e., TAPSE and TAPSE/estimated pulmonary arterial systolic pressure, PASP) and peak oxygen consumption (peak VO_2_) from baseline to the 36-months visit (or the last available before the outcome) in the 3 groups.

### Study procedures

Study procedures have been previously published in detail [10, 11]. In brief, blood samples were collected by venipuncture after overnight fast. To obtain serum and plasma, samples were centrifuged within 30 min, frozen, and stored at – 80 °C until assayed. Brain natriuretic peptide levels were assessed using a point-of-care device (RapidPIA™, Sekisui Medical Co, Tokyo, Japan) in a dedicated core-lab (John and Lucille van Geest Biomarker Facility, University of Leicester, UK). The impairment of the Insulin axis has been described as the diagnosis of Type 2 diabetes mellitus (T2D) following guidelines or HomeOstasis Model Assessment (HOMA) greater than 2.5 (HOMA = insulin (mcU/ml) × glucose (mmol/l)/22.5.

#### Echocardiographic study

A complete transthoracic echocardiographic study, including complete M-mode, 2-dimensional, and Doppler analyses was performed at baseline and after a mean follow-up of 36 months, following the American Society of Echocardiography and European Society of Cardiovascular Imaging guidelines recommendation.

All measures were performed with the patients in the lateral recumbent position, and images were obtained by standard parasternal (short and long axis) and apical views. Echocardiographic exams were performed by expert trained physicians in each center, and data were revised in blind by two independent expert physicians of the core center according to previously published methods [10, 11].

#### Cardiopulmonary exercise test

All patients underwent an incremental symptom-limited cardiopulmonary exercise test (CPET) on a bicycle ergometer. After a 1-min warmup period at 0-W workload, a ramp protocol of 10 W/min was started and continued until limiting symptoms or other indications for exercise termination appeared [4, 8]. Respiratory gas exchange measurements were obtained breath-by-breath using a commercially available system (Vmax 29C, Sensormedics, Yorba Linda, California). VO_2_ was recorded as the mean value of VO_2_ during the last 20 s of the test. The ventilatory anaerobic.

threshold was detected using the V-slope method. The ventilation per min (VE) versus carbon dioxide production (VCO2) relationship was measured by plotting ventilation against VCO2 obtained every 10 s of exercise (VE/VCO2 slope). The VE/VCO2 slope was calculated as a linear regression function, excluding the nonlinear part of the relationship after the onset of acidotic drive to ventilation.

### Statistical analysis

Normally distributed continuous variables were expressed as mean ± standard deviation (SD), whereas continuous data with skewed distributions were expressed as median [interquartile range (IQR)]. Categorical variables were expressed as counts and percentages. The distribution of the variables was tested with the Kolmogorov–Smirnov test.

Normally distributed variables were compared between groups using the two-sided, unpaired Student’s t-test, assuming unequal variance. Non-normally distributed variables were compared between groups using the nonparametric Mann–Whitney U-test or the Kruskal- Wallis test. Rates and proportions were compared between groups of interest using the chi-square test or correction for continuity test. For continuous variables normally distributed, statistical comparisons between groups were established by carrying out the one-way ANOVA test. P-values from the analysis of variance were adjusted using the Holm approach. When the ANOVA test revealed a statistical difference, pairwise comparisons were made by Tukey’s HSD (Honestly Significant Difference) test. Difference between groups and continuous VO_2_max levels were assessed through the ANCOVA model, considering all the baseline variables resulting statistically different between groups (e.g., age, gender, time from diagnosis). Age sex, NYHA class, BMI, electrolytes, and clinical or biological plausible variables were tested in a univariate analysis and used as a covariate in the ANCOVA model or used to calculate a propensity score if more than 5. Moreover, the relationship between VO_2_max and HOMA-IR (including the presence and absence of diabetes) was explored through Pearson or Spearman coefficient. A linear regression model was also provided, together with the related fitting curve. In order to test if there’s a relationship between HOMA-IR classes (i.e., no diabetes, I tertile of IR, II tertile of IR, III tertile of IR and presence of diabetes) and VO_2_ max, a logistic regression model was fitted providing the odds ratios and 95% confidence interval, considering as reference class both the absence and the presence of diabetes. P-values < 0.05 were considered statistically significant. All data were analysed using R version 3.0 (http://www.r-project.org).

## Results

From the original cohort of 525 patients, complete data about insulin impairment were available for 480 patients; as a result, these patients represent the baseline population of the present analysis.

### Demographic characteristics

Baseline demographic characteristics of the final cohort are depicted in Table 1. Overall, 308 (64%) patients displayed an impairment of the insulin axis. Specifically, 120 patients were affected with T2D (25% of the total population) and 188 patients displayed IR (39% of the total population). Both IR and T2D patients displayed a significantly higher BMI compared with EU (26 ± 4, 30 ± 5, and 30 ± 6 respectively, p < 0.001), and had more frequently ischemic heart disease as aetiological cause of HF. As expected, T2D patients had higher glycaemia and glycosylated-haemoglobin (HbA1C) levels when compared with IR and EU patients (p < 0.01). T2D and IR had comparable insulinemia levels, significantly higher than euglycemic patients, while HOMA Index was higher in T2D compared with both IR and EU.

**Table 1:** Clinical Characteristics of the CHF population classified as Euglycemic, IR, and DM

Characteristics	Study cohort (n = 480)	Euglycemic (n = 172)	IR (n = 188)	T2D (n = 120)	Pearson’s Chi-squared	ANOVA F-value	Kruskal–Wallis *Chi-squared*	p-value
Age (year)	62.1 ± 12.2	62.5 ± 14.8	59.8 ± 11.3	64.7 ± 9.4^§^		6.1		< 0.01
Sex (male)	356 (74%)	117 (68%)	137 (72%)	102 (85%)	0.2			0.9
NYHA class n (%)					5.1			0.3
I	43	19 (13.5%)	17 (10.8%)	7 (5.9%)				
II	224	73 (51.8%)	88 (55.7%)	63 (52.9%)				
III–IV	144	49 (34.7%)	53 (33.5%)	49 (41.2%)				
Ischemic/non ischemic aetiology	220/197	56/84	82/37	82/76	21.6			< 0.001
Year of disease	11 [6–17]	12 [6–17]	10 [6–17]	11 [7–18]			5.5	0.06
Systolic blood pressure (mm/Hg)	120 ± 18	118 ± 17	118 ± 17	122 ± 19^§^		1.9		0.15
Diastolic blood pressure (mm/Hg)	74 ± 10	74 ± 12	74 ± 9.3	74 ± 9.8		0.1		0.9
BMI (kg/m^2^)	28 ± 5.4	26 ± 4	30 ± 5*	30 ± 6*		27.3		< 0.001
eGFR (ml/min/1.73 m^2^)	87 ± 41	79 ± 37	95 ± 42*	87 ± 43		6.0		< 0.01
Current smokers (%)	15.4	14.1	16.2	15.7	0.4			0.8
NT pro BNP (pg/ml)	935 [319–2822]	1134 [387–3580]	972 [200–2456]	952 [383–2824]			2.5	0.15
EF (%)	31.7 ± 7.2	31.5 ± 8.1	32.0 ± 7.7	31.6 ± 7.0		0.46		0.8
Atrial fibrillation n (%)	64	32 (24.1)	20 (28.1)	12 (20.4)	1.25			0.53
ICD (%)	213	65 (44.2)	82 (50)	66 (53.2)	2.3			0.3
CRT (%)	69	20 (13.7)	28 (17.1)	21 (16.9)	0.8			0.7
Glycemia (mg/dl)	113 ± 40	92 ± 13	108 ± 28*	145 ± 52*^,§^		86.3		< 0.001
Glycosylated hemoglobin (%)	6.3 ± 1.3	5.7 ± 0.5	6 ± 0.9	7.5 ± 1.6*^,§^		80.8		< 0.001
Insulinemia (microU/l)	12.9 (8.4–24.6)	6.9 (5.1–9.3)	18.4 (14.2–32.4)*	17.0 (11.1–38.7)*		193.55		< 0.001
HOMA-IR	3.5 (2.0–6.4)	1.7 (1.2–2.1)	4.8(3.5–8.0)*	5.3(3.5–13.7)*			254.1.01	< 0.001
THERAPY								
Insulin (%)	–	0	0	51 (13.6%)				
Antidiabetics (%)	–	0	0	72 (19.2%)				
Insulin and antidiabetics (%)	–	0	0	17 (4.6%)				
B-blocker (%)		135 (91.8)	117 (93.4)	156 (92.8)	0.3			0.8
ACE-I/ARBs (%)		90 (61.2)	101 (60.5)	73 (57.9)	0.3			0.8
MRA (%)		78 (53.1)	84 (50.3)	65 (52)	0.2			0.9
Diuretics (%)		104 (70.7)	136 (81.4)	103 (81.7)	6.7			< 0.05
Amiodarone (%)		38 (25.8)	36 (21.5)	22 (17.6)	2.7			0.3
Digoxin (%)		18 (12.2)	18 (10.8)	14 (11.1)	0.2			0.9
Antiplatelets (%)		53 (36.0)	59 (35.3)	41 (32.5)	0.4			0.8
Antithrombotic (%)		80 (29.0)	103 (37.4)	92 (33.4)	10.1			< 0.05
Lipid-lowering medications (%)		72 (49.0)	108 (64.7)	95 (76.0)	21.5			< 0.001
Ivabradine (%)		16 (10.9)	34 (20.3)	21 (16.8)	5.2			0.07

NYHA, New York Heart Association; BMI, body mass index; eGFR, estimated glomerular filtration rate (CKD-EPI); NT pro BNP, N-terminal proB-type natriuretic peptide; EF, ejection fraction; ICD, implantable cardioverter-defibrillator; CRT, cardiac resynchronization therapy; HOMA, HomeOstasis Model Assessment; ACE-I, angiotensin-converting-enzyme inhibitors; ARBs, angiotensin-receptor blockers; MRA, Mineralocorticoid receptor antagonists

^*^p < 0.05 respect Euglycemic

^§^p < 0.05 respect IR

No differences were found regarding sex, duration of disease, NYHA classes, current smoke habitus, NT-proBNP, and HF treatment except diuretics, which were employed less frequently in euglycemic patients (p < 0.05).

### Echocardiography

Baseline echocardiographic findings are depicted in Table 2. T2D patients, compared with other groups, displayed a significantly increased IVS thickness (11 ± 2 mm, 10 ± 2 mm, 10 ± 2 mm, T2D, IR, and EU respectively, < 0.05) and LV mass and a higher relative wall thickness (0.34 ± 0.1, 0.32 ± 0.1, and 0.32 ± 0.1 T2D, IR, and EU respectively, p < 0.05), indicating less eccentric remodelling. Such LV architectural alterations in T2D patients were paralleled by worse LV filling dynamics, suggested by both larger left atrial volume index (48 ± 26 ml/m^2^ vs 43 ± 19 ml/m^2^ and 38 ± 17 ml/m^2^, p < 0.01), and particularly by a higher E/e′ ratio (16 ± 9, 14 ± 8, and 12 ± 6 respectively, p < 0.01) when compared with both IR and EU patients, respectively. Indexes of systolic RV function (TAPSE and RFAC) are not different between the three groups (Table 2); indexes of right ventricular-pulmonary arterial uncoupling were equally more impaired in T2D compared with IR and EU. Indeed, a lower TAPSE/PASP ratio was found in T2D patients (0.52 ± 0.2, 0.6 ± 0.3, and 0.6 ± 0.3, p < 0.05). Likewise, increased right atrial volumes (34 ± 19 ml/m^2^ vs 30 ± 14 ml/m^2^ and 26 ± 13 ml/m^2^, p < 0.05) as well as higher percentage of moderate/severe TR were recorded in T2D patients compared with both IR and EU patients. In addition, when T2D patients were compared with regard to glycosylated-hemoglobin levels (< 7% n = 76, 7–8% n = 23, and > 8% n = 21 respectively), despite displaying similar values with regard to indexes of systolic RV function, patients with higher HbA1C displayed a more frequent RV-AP impairment, as testified by an impaired TAPSE/PASP ratio [35%, 36% and 57%, respectively—X^2^ (2, N = 120) = 7.6353; p = 0.02].

**Table 2:** Echocardiographic characteristics of the whole CHF population classified as Euglycemic, IR, and DM

Characteristics	Study cohort	Euglycemic (n = 172)	IR (n = 188)	T2D (n = 120)	ANOVA F-value	p-value
IVSd (mm)	10.6 ± 2	10 ± 2	10 ± 2	11 ± 2*^§^	6.1	< 0.05
LVEDd (mm)	62.7 ± 8.4	63.2 ± 8.4	62.9 ± 8.8	62.0 ± 8.0	0.4	0.6
PWd (mm)	9.6 ± 1.5	9.5 ± 1.5	9.6 ± 1.5	9.8 ± 1.6	1.3	0.3
LVEDVi (ml/m^2^)	97.8 ± 38.2	99.1 ± 33.6	100 ± 43.2	93.2 ± 36.4	1.1	0.3
RWT (IVSd + PWd)/LVEDd	0.33 ± 0.1	0.32 ± 0.1	0.32 ± 0.1	0.34 ± 0.1^§^*	4.7	< 0.05
LVMi	145 ± 44	139 ± 29	146 ± 37	150 ± 85	1.4	0.3
LAVi (ml/m^2^)	42.7 ± 21.3	38.3 ± 17.2	43.0 ± 19.0	48.0 ± 26.4*	5.8	< 0.01
E velocity (cm/sec)	73.2 ± 26.0	73.3 ± 26.2	69.9 ± 22.0	77.5 ± 30.0^§^	2.1	0.12
E/e′	14 ± 8	12 ± 6	14 ± 8*	16 ± 9^§*^	6.1	< 0.01
PASP (mmHg)	37 ± 14.5	38 ± 15	35 ± 15	39 ± 15	2.4	0.1
TAPSE	18.7 ± 4.6	19.1 ± 4.7	18.7 ± 4.6	18.2 ± 4.5	1.4	0.2
TAPSE/PASP	0.6 ± 4.6	0.6 ± 0.3	0.6 ± 0.3	0.52 ± 0.2^§^*	3.2	< 0.05
Moderate/severe tricuspid regurgitation (n; %)	112; 23	36; 21	41; 22	35; 29	4.2	< 0.1
RVDd (mm)	36.3 ± 9.5	37.3 ± 7.6	36.5 ± 11.2	34.9 ± 9.0	0.8	0.4
RVFAC (%)	55 ± 12	56.5 ± 10.7	55.9 ± 11.6	53.5 ± 13,5	1.6	0.5
RADVi (ml/m^2^)	30 ± 16	26 ± 13	30 ± 14	34 ± 19*	4.0	< 0.05

IVSd, Inter Ventricular Septum Diastole; LVEDd, Left Ventricular End Diastolic Diameter; PWd, Posterior Wall Diastole; LVEDVi, LVEDV/BSA (Left Ventricular End Diastolic Volume/BSA); RWT, Relative Wall Thickness; LVMi, LVM/BSA (Left Ventricular Mass/BSA); LAVi, LAV/BSA (Left Atrial Volume/BSA); E Velocity; PAPS, Pulmonary Artery Systolic Pressure; TAPSE, Tricuspid Annular Plane Systolic Excursion; RVDd, Right Ventricular Diastolic Diameter; RVFAC, Right ventricular fractional area change; RADVi, RADV/BSA (Right Atrial Diastolic Volume/BSA)

^*^p < 0.05 respect Euglycemic

^§^*p* < 0.05 respect IR

**Fig. 1:**
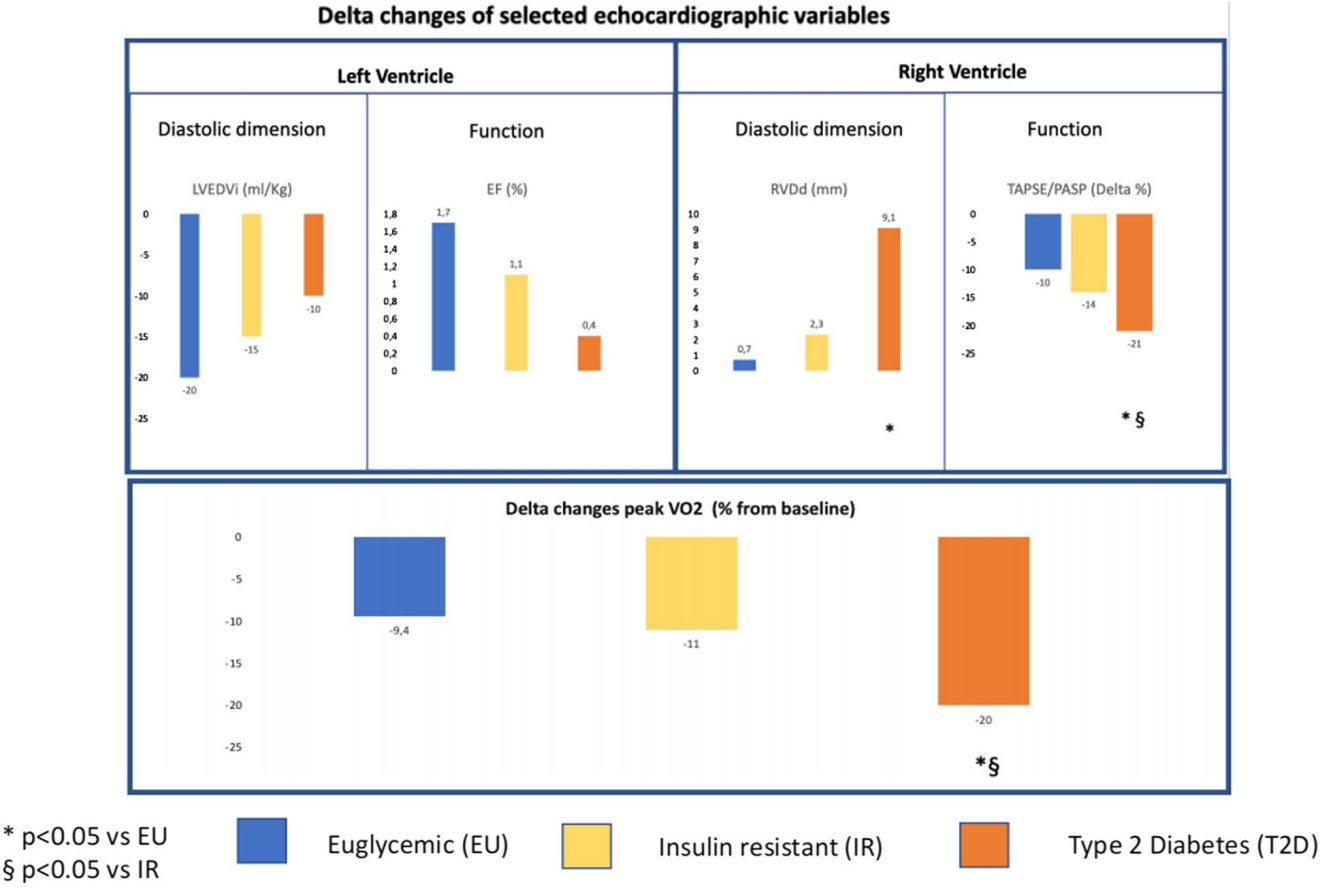
Longitudinal changes of Left and Right Ventricular architecture and function (**A**) and exercise capacity (**B**) of patients grouped with regard to insulin action impairment. Delta changes of selected variables of left ventricle (LV) and right ventricle (RV) architecture and function from baseline at 36 months (**A**). Whereas delta changes of LV parameters did not significantly differ between three groups, T2D patients displayed a more prominent progression of RV parameters. This phenomenon is paralleled by a more important impairment in cardiovascular performance, as testified by the delta change of peak VO_2_ from baseline (**B**)

Particularly intriguing were the longitudinal data (Fig. 1). The median time between Exam 1 and Exam 2 was 3.0 years (IQR: 2.1–3.3). Only minor changes were observed with regard to LV architecture and function in the three groups over time, while RV structure and function worsened significantly in the T2D patients compared with IR and EU groups. As a prototype, only slight changes among the three groups were observed over time in LV-EF (+ 0.4%, + 1.1%, + 1.7%, DM, IR, and EU respectively, p = 0.43) and LVEDVi (− 10 ml/Kg, − 15 ml/Kg, and − 10 ml/Kg), while RV dimensions increased by 26% in the TD2 group, and RV dysfunction and uncoupling progressed to a larger extent particularly in the TD2 group, as testified by percent delta changes of the TAPSE/PASP ratio (− 21%, − 14%, and − 10%, respectively, p < 0.05) (see Fig. 1A). Notably, the TAPSE/PASP ratio was equally influenced by a decrease in the TAPSE value paralleled by an increase in the PASP, suggesting that T2D patients displayed an RVAUC impairment *as a whole,* more than the progressive impairment of a single factor.

### Cardiopulmonary performance

Baseline exercise capacity was more compromised in T2D patients when compared with both euglycemic and IR patients, as shown by a significant lower distance on the 6-min walking test (352 ± 93 m, 386 ± 107 m, and 397 ± 101 m, DM, IR, and EU respectively, p < 0.01), (Table 3). Measures of cardiopulmonary performance are depicted in Table 4. Congruent with the echocardiographic data, T2D patients displayed a significant lower peak oxygen consumption when compared to IR and EU patients (respectively, 15.8 ± 3.8 ml/Kg/min, 16.5 ± 4.3 ml/Kg/min, and 18.4 ± 4.3 ml/Kg/min, p < 0.003). No significant differences were observed with regard to VE/VCO_2_ slope. A logistic regression model was performed to test the relationship between IR class and peak VO_2_ considering as reference class both the absence (euglycemic) and the presence of diabetes (OR; [95% CI], p-value) (0.89; [0.8–0.98], p < 0.05) and (1.12; [1–1.3], p = 0.05) respectively. Our results showed a relationship between HOMA-IR classes (i.e., euglycemic, IR, and T2D) and peak VO_2_. Finally, an ANCOVA model was performed, considering all the baseline variables resulting statistically different between groups (i.e., age, BMI, and aetiology), showing peak VO_2_ as an independent variable even after the adjustment for the covariates between the three groups (F-value 4.03, p < 0.05).

**Table 3:** Results of the Six-minute walking test distance of the whole CHF population classified as Euglycemic, IR, and DM

Characteristics	Study cohort	Euglycemic	IR	T2D	ANOVA F-value	Kruskal–Wallis *Chi-squared*	p-value
Heart Rate (bpm) (T0)	68 [60–75]	65 [60–73, 5]	68 [62–77]*	72 [64–76]*	5.5		< 0.01
sPO_2_ (%) (T0)	97 [96–98]	97 [95–98]	97 [96–98]	97 [96–98]	0.5		0.6
Fatigue Borg Scale (T0)	0 [0–1]	0 [0–0.6]	0 [0–1]	0 [0–1]	–	1.8	–
Distance (m)	380 ± 102	397 ± 101	386 ± 107	352 ± 93*^,§^	3.6		< 0.01
sPO_2_ MAX (%)	98 [97–99]	98 [97–99]	98 [97–98]	98 [97–98]	0.8		0.45
sPO_2_MIN (%)	96 [94–97]	95 [94–97]	96 [94–97]	96]94–97]	0.3		0.8
HR MAX (bpm)	85 [75–97.2]	84 [73–98]	87 [76–97]	85 [77–94]	0.9		0.4
Heart Rate (end) (bpm)	80 [70–90]	76 [68–86.5]	80 [70–93]	81 [72–92]	2.3		0.1
sPO_2_ (end) (%)	97 [95–98]	97 [95–98]	97 [96–98]	97 [95–98]	0.3		0.7
Fatigue Borg Scale (End)	2 [1–3]	1 [0.5–3]	2 [1–4]	2 [1–3]		3	0.2

^*^p < 0.05 respect Euglycemic

^§^*p* < 0.05 respect IR

**Table 4:** Cardiopulmonary Exercise Test parameters of the whole HF population classified as Euglycemic, IR, and DM

Characteristics	Study cohort	Euglycemic	IR	T2D	ANOVA	
					F-value	*p*-value
VO_2_AT(ml/kg/min)	10.7 ± 3.5	11.4 ± 3.0	10.9 ± 3.6	9.5 ± 3.6	2.2	0.1
VO_2_max (ml/kg/min)	16.3 ± 4.9	18.0 ± 4.3	16.5 ± 4.3*	15.8 ± 3.8*	5.5	< 0.01
VE-VCO_2_SLOPE	32.3 ± 6.2	31.5 ± 6.2	32.3 ± 6.4	33.4 ± 7.8	0.8	0.5

VO_2_AT, oxygen consumption at anaerobic threshold; VO_2_max, Maximal oxygen uptake or consumption; VE-VCO_2_SLOPE, minute ventilation/carbon dioxide production slope

^*^p < 0.05 respect Euglycemic

^§^*p* < 0.05 respect IR

Longitudinal changes in cardiopulmonary performance were also intriguing (Fig. 1B), pointing to a more rapid deterioration in diabetic patients vs. insulin resistant and euglycemic subjects. Specifically, 30% of the final cohort, 144 patients, evenly distributed across the groups (n = 44 EU, 53 IR, 47 DM) performed a follow-up CPET. While the decline in maximal oxygen consumption was only marginal in IR and EU groups (− 10 and − 11%), T2D patients displayed a more pronounced worsening of the cardiopulmonary performance, that reached—20% (p < 0.01; − 3.2 ml/min/Kg, −  1.91 ml/min/Kg, and − 1.74 in T2D, IR and EU, respectively) (Fig. 1B). No differences were observed with regard to VE/VCO_2_ slope between groups (data not shown).

### Outcomes

As elsewhere described [11], T2D and IR were associated with poor outcome [HF 1.34 (1.03–1.73), p = 0·03]; however, when considered separately, T2D and not IR patients showed a significant association with the primary endpoint, reaching 70% of the patients with T2D and 52% in the patients without T2D (p = 0.001). Interestingly, among diabetic patients, those displaying a greater decline in CPET performance (i.e., peak VO_2_) and right ventricular pulmonary arterial coupling (i.e., TAPSE/PASP) were burdened by a worse prognosis. Indeed, overall, patients with delta changes > 10% in CPET, and TAPSE/PAPS also displayed a worse prognosis (as a prototype, maximal oxygen consumption delta changes > 10%: OR: 3.4 (1.9–4.1) p < 0.01). Notably, patients with a greater impairment of RV dynamics were significantly more common in the T2D group than in the IR and EU group. For example, with regard to peak oxygen consumption, respectively, 80%, 39%, and 30% of patients displayed delta change > 10% in T2D, IR and EU, respectively [X^2^(2 N = 144) = 25.97, p < 0.01].

## Discussion

The present report sheds new light on the differential role of insulin resistance and hyperglycaemia in the natural history of heart failure, as well as on the pivotal role of right ventricular architecture and dynamics. Several are the principal findings herein described: (a) T2D rather than IR further impairs left and right chambers morphology and function in HF, as well as cardiopulmonary performance, as shown by a broad variety of echocardiographic and CPET indexes; (b) T2D equally heavily impacts on longitudinal changes in cardiac morphology and function, and exercise capacity suggesting the concept that hyperglycaemia rather than IR induces further pathologic remodeling and exercise impairment; and (c) the rate of deterioration of RV architecture and function over time is associated with a worse prognosis, underlying the pivotal role of RV dynamics in HF progression.

To the best of our knowledge, the present study represents the most comprehensive investigation dwelling upon longitudinal changes of right and left cardiac morphology and function combined with cardiopulmonary performance comparing HF patients with regard to T2D, IR and EU status. Further, these data enhance the understanding of how T2D acts as an accelerator of disease progression in patients with HF.

### Baseline echocardiographic and cardiopulmonary performance findings

Longitudinal population-based data have demonstrated that IR predicts worsening of LV function and remodeling when compared to euglycemic patients [13]. Further, in the Treatment Options for Type 2 Diabetes Mellitus in Adolescents and Youth (TODAY) study it was shown that T2D patients displayed a greater diastolic function decline when compared to non-diabetic patients [14]. Finally, in the Atherosclerosis Risk In the Community (ARIC) Study, it has been reported that dysglycaemia was associated with subtle and subclinical alterations of cardiac structure, with impaired left ventricular systolic and diastolic function [15]. In HF patients, T2D is associated with adverse structural and functional cardiac remodeling [16]. In our report, T2D patients showed a significantly worse LV remodeling in respect to both EU and IR, characterized by a higher LV septum thickness, LV mass, and higher relative wall thickness, associated with a higher E/e’ ratio indicating higher filling pressures. Of note, since these alterations are not present in EU and IR groups, our data suggest that hyperglycemia, rather the IR, affects heart architecture and function in HF patients.

Although neglected in the past, mounting evidence is congruent on the pivotal role of right heart in driving prognosis in HF, especially when the backward transmission of LV filling pressures rises up to generating an increased RV afterload [17]. A critical event appears the loss of left atrial capacitance/conductance capability. When the right ventricle faces an increase in afterload, it tends to adapt by increasing its contractility (coupling) to ensure appropriate pulmonary perfusion [18]. When this compensation mechanism fails (uncoupling), HF patients become highly symptomatic and display a poor prognosis [18]. In our cohort, T2D patients displayed increased LV filling pressures, as showed by higher E/e′ ration and larger LA volumes. Interestingly, even if indexes of systolic RV function (TAPSE and RFAC) are not different between the three groups—in line with previous studies showing that T2D does not impact on RVFAC [19]—T2D patients showed a more compromised TAPSE/PASP ratio, which is an easily assessable echocardiographic index that has recently showed a good correlation with the invasively assessed RV-PA coupling [20]. This parameter has already showed to be strongly associated with increased mortality in HF patients [21–23]. Our results pointed out a major role of the RV-PA coupling on the single indexes of RV function, further supporting the concept that TAPS/PASP ratio to be impaired in HF. Last but not least, T2D patients displayed also larger RA volumes, which is a signal for increased RV diastolic dysfunction, which usually appears before systolic dysfunction become overt, and a higher percentage of moderate-to-severe TR, also indicating worse right chambers dynamics [24].

Congruent with our results, serum insulin was inversely associated with right ventricle function and lung volumes in the general population suggesting that increased insulin levels may contribute to subclinical cardiopulmonary circulation impairment [25] in HF patients. In addition, when T2D patients were compared with regard to HbA1C levels, the RV-PA coupling resulted significantly more frequently impaired in patients with higher HbA1C levels, further pointing out the important role of hyperglycemia in HF. This was in line with previous studies [26], in which patients with HbA1C > 7% were more likely to develop RV dysfunction, supporting the role of hyperglycemia on HF [27], with the known effects on microvascular dysfunction [28] and on coronary atherosclerosis [29].

T2D has been associated with lowered peak VO_2_ in the general population [30, 31] and HF patients [32]. Notably, recent studies showed that although there was no significant difference in peak cardiac output, peripheral extraction was lower in patients with T2D compared to controls. In our cohort, we confirm that T2D patients displayed a greater cardiopulmonary impairment, as testified by a significant reduction of peak VO_2_ when compared to EU patients.

### Longitudinal echocardiographic and cardiopulmonary performance findings and impact on outcomes

As elsewhere described [11], the T.O.S.CA. Registry showed that the impairment of the insulin action (i.e., patients with abnormal HOMA index or T2D) is associated with worse outcome. However, when the presence of IR or T2D were investigated alone, only T2D was associated with the primary endpoint, but not IR alone. These findings are not surprising, considering that whereas T2D has been unequivocally shown as a strong predictor of mortality in HF patients, with a more preponderant role in women [33], and no difference among the HF spectrum [34], few and inconsistent results are available in the literature with regard to IR. Indeed, if on the one hand IR has been proven to be a predictive factor associated with poor outcome in HF [35], on the other hand, wider and more recent investigations showed no association between prediabetes and incident HF [36].

As a possible explanation, in the T.O.S.CA. Registry the entity of changes of cardiac structure and function diverged in T2D and IR patients. Specifically, delta changes from baseline of right ventricle diameters and index of ventricular-arterial uncoupling were significantly larger in T2D patients compared to IR and EU, pointing to a rapid deterioration of right chambers architecture and dynamics. Notably, this was paralleled by a significant larger delta change in peak VO_2_, testifying a higher degree of worsening of the cardiovascular performance in T2D patients when compared to the other groups.

Congruent with these results, recent evidence showed that HFpEF patients displayed a greater impairment over time of right ventricular structure and function compared to left ventricle function [37]. On the other hand, in the same population [37], it has been unequivocally proven that patients developing RV dysfunction had a worse outcome. Taken altogether, our findings strongly may point to a pivotal role of right ventricle as a potential key player of the poorer outcome of HF-T2D patients shown in the T.O.S.CA. Registry and other larger cohort studies.

### Type 2 diabetes impacts dramatically on right heart performance, exercise capacity, and accelerates their worsening over time

According to cross-sectional data from the current analysis, while systolic performance was impaired to a similar extent among groups, T2D patients showed a profile characterized by greater LV concentric remodeling, with increased filling pressures (E/e′). This alteration in LV dynamics may be attributable in the T2D-CHF patient to a metabolic shift from glucose consumption to free fatty acids, highly unfavorable from an energetic point of view [38]. Such an increase in LV filling pressures is paired with larger atrial sizes in T2D patients and may reflect a parallel increase in atrial pressure with potential reverberation on the pulmonary circulation, a condition commonly found in diabetes [39, 40]. A recently published study performed on biopsies of patients undergoing cardiac surgery demonstrated that left atrium of T2D-patients displayed greater stiffness and reduced contractile forces mainly due to a worse calcium metabolism [41]. Backward transmission of increased filling pressure from the left heart combined with loss of compliance and dysfunction of the pulmonary microcirculation in the T2D patient are likely to lead to right heart chamber overload [42]. According to our data, patients with T2D showed worse TAPSE/PASP ratio, which may represent a good surrogate of right ventricular arterial coupling, given its good correlation with its invasively assessment [43]. Right ventricular-arterial uncoupling underlies clinical worsening in all conditions rooted on right ventricular overload [18] and is a powerful independent predictor of HF mortality [17]. Taken together, the concurrent presence of diabetes in HF patients constitutes a serious aggravation of cardio-respiratory dynamics. In fact, it is not surprising to find a worse peak oxygen consumption in such patients, as this important parameter of exercise capacity correlates more with RV than LV function [17]. Interestingly, our data also pinpoint that T2D patients are more prone to have a more rapid deterioration of right ventricular-pulmonary arterial uncoupling, right heart size and exercise capacity, in line with previous studies [26]. This could lead to the speculation that the low- grade inflammation present in the diabetic patients constitutes a long-standing detrimental factor in the HF natural history catalysing its progression to poorer outcomes [44].

### Clinical impact

The findings of the present study further support the concept that patients with HF should undergo a systematic metabolic evaluation, focused to prevent or delay the onset of insulin resistance (i.e., prediabetes) or hyperglycemia (i.e., T2D) with specific interventions (e.g., nutrition, physical activity, specific drugs). Furthermore, considering the worse LV and RV remodeling and dynamics and exercise performance of T2D patients and their rapid deterioration over time, this subset of patients should probably receive more aggressive therapeutic interventions. Indeed, our results showed that patients with a poorer glycemic control (i.e., higher HbA1c) displayed a more frequent impairment of RV-PA coupling, pointing out a preponderant role of hyperglycemia (i.e., T2D) rather than insulin resistance on cardiovascular impairment. In line with this hypothesis, intriguingly, it has been demonstrated that drugs acting on insulin resistance, (i.e., glitazones), were non-effective or deleterious in clinical and experimental studies on HF, whereas glucose lowering drugs with no direct effect on insulin sensitivity [i.e., sodium-glucose co-transporter-2 (SGLT2) inhibitors] have recently proved to be effective in reducing mortality and hospitalization in patients with HF [45, 46]. Indeed, in the last HF guidelines, these drugs entered with the highest level of evidence and recommended with an ACE-I/ARNI, a beta-blocker and an MRA, for patients with HFrEF [47, 48], and our results further support an important role of T2D control and management in HF patients.

On the other hand, our data showed a pivotal role of the right ventricle in determining the poor prognosis of T2D patients in HF, with RV-PA uncoupling as key-player of the more impaired cardiovascular performance and of the poorest outcome displayed by these patients in the T.O.S.CA. registry [11]. This is in line with the hypothesis that exercise capacity in HF patients is more closely related to RV than LV performance [17, 49], suggesting the need for evidence-based management strategies targeting RV dysfunction in HF, as promising objective of future investigations. Fascinatingly, data on the effects of sodium-glucose cotransporter 2 inhibitors in patients demonstrating signs of RHF but not LV impairment are lacking, and this should be addressed by future research.

In conclusion, compared to patients without diabetes, heart failure patients with type 2 diabetes display a higher degree of progressive right ventricular dysfunction and exercise impairment, associated with a poorer outcome. Findings from the T.O.S.CA. Registry shed light upon the key role of the right ventricle in HF, which appears a pivotal player in underlying the poor outcome faced by T2D HF patients.

### Study limitations and strengths

The observational nature of the current study precludes the elucidation of the putative biological mechanism(s) underlying the progressive right ventricular dysfunction and exercise impairment in patients with heart failure and diabetes mellitus [50]. Despite representing the gold standard technique for investigating RV morphology and function [51], cardiac magnetic imaging was not performed for all patients. However, the T.O.S.CA. Registry represents a snapshot of real world, with echocardiography as the most available and feasible technique in the clinical practice. Another limitation is the relatively small sample-size of the T.O.S.CA. Registry. However, this is one of the few investigations measuring insulin serum concentrations and HOMA index, investigating the entire spectrum of insulin impairment in a HF population. Finally, the availability of a complete echocardiographic evaluations as well as of measures of cardiopulmonary performance at different time points represent another strength.

## Data Availability

All data generated or analyzed during this study are included in this published article

## Appendix

The T.O.S.CA. investigators include: Cittadini A, Marra AM, Arcopinto M, D’Assante R, Saccà L, Monti MG, Napoli R, Matarazzo M, Stagnaro FM, Piccioli L, Lombardi A, Panicara V, Flora M, Golia L, Faga V, Ruocco A, Della Polla D, Franco R, Schiavo A, Gigante A, Spina E, Sicuranza M, Monaco F, Apicella M, Miele C, Campanino AG, Mazza L, Abete R, Farro A, Luciano F, Polizzi R, Ferrillo G, De Luca M, Crisci G, Giardino F, Barbato M (Department of Translational Medical Sciences, Federico II University, Naples, Italy); Salzano A, Ranieri B (IRCCS S.D.N., Naples, Italy); Bossone E (AORN A Cardarelli, Naples, Italy); Ferrara F, Russo V, Malinconico M, Citro R (Heart Department, Cardiology Division, "Cava de' Tirreni and Amalfi Coast" Hospital, University of Salerno, Salerno, Italy); Guastalamacchia E, Iacoviello M, Leone M, (University of Bari “Aldo Moro”, Bari. Italy); Triggiani V, Giagulli VA (Interdisciplinary Department of Medicine-Section of Internal Medicine, Geriatrics, Endocrinology and Rare Diseases. University of Bari "A. Moro", Bari, Italy) Cacciatore F, Maiello C, Amarelli C, Mattucci I (Heart Transplantation Unit, Monaldi Hospital, Azienda Ospedaliera dei Colli, Naples, Italy); Limongelli G, Masarone D, Calabrò P, Calabrò R, D’Andrea A, Maddaloni V, Pacileo G, Scarafile R (Cardiology SUN, Monaldi Hospital, Azienda Ospedaliera dei Colli, Second University of Naples, Naples, Italy); Perticone F, Belfiore A, Sciacqua A, Cimellaro A (University Magna Graecia of Catanzaro, Catanzaro, Italy); Perrone Filardi P, Casaretti L, Paolillo S, Gargiulo P (Department of Advanced Biomedical Sciences, Federico II University of Naples, Naples, Italy); Mancini A, Favuzzi AMR, Di Segni C, Bruno C, Vergani E (Operative Unit of Endocrinology, Catholic University of the Sacred Heart, Rome); Volterrani M, Massaro R (IRCCS S. Raffaele Pisana, Roma, Italy); Vriz O, Grimaldi F (Azienda Ospedaliero-Universitaria “Santa Maria della Misericordia” San Daniele del Friuli, Udine, Italy); Castello R, Frigo A (Azienda Ospedaliera Universitaria Integrata di Verona, Italy); Campo MR, Sorrentino MR (Ospedali Riuniti di Foggia, Italy); Modesti PA, Malandrino D (Università di Firenze, Italy); Manfredini R, De Giorgi A, Fabbian F (Azienda Ospedaliera-Universitaria S. Anna, Ferrara, Italy); Puzzo A, Ragusa L (I.R.C.S.S. Oasi Maria SS, Troina, Italy.); Caliendo L, Carbone L (Ospedale Santa Maria della Pietà, Nola, Napoli, Italy); Frigiola A, Generali T, Giacomazzi F, De Vincentiis C, Ballotta A (IRCCS San Donato Milanese, Milano, Italy); Garofalo P, Malizia G (Ospedali Riuniti "Villa Sofia – Cervello”, Palermo, Italy); Milano S, Misiano G (Policlinico P. Giaccone, Palermo, Italy); Suzuki T, Israr MZ, Bernieh D, Cassambai S, Yazaki Y (Department of Cardiovascular Sciences and NIHR Leicester Biomedical Research Centre, University of Leicester, Glenfield Hospital, Leicester, UK); Heaney LM (School of Sport, Exercise & Health Sciences, Loughborough University, Loughborough, UK); Eagle KA (Michigan Frankel Cardiovascular Center, University of Michigan, Ann Arbor, Michigan); Ventura HO (John Ochsner Heart and Vascular Institute, Ochsner Clinical School-the University of Queensland School of Medicine, New Orleans, Louisiana, USA); Colao A (Clinical Medicine and Surgery Department—Federico II University, Naples, Italy); Bruzzese D, Statistical Management (Department of Public Health, University Federico II of Naples, Naples, Italy).
